# Androgen-regulated genes differentially modulated by the androgen receptor coactivator L-dopa decarboxylase in human prostate cancer cells

**DOI:** 10.1186/1476-4598-6-38

**Published:** 2007-06-06

**Authors:** Katia Margiotti, Latif A Wafa, Helen Cheng, Giuseppe Novelli, Colleen C Nelson, Paul S Rennie

**Affiliations:** 1 Department of Pathology and Laboratory Medicine, Faculty of Medicine, University of British Columbia, Vancouver, BC, V6T 2B5, Canada; 2 The Prostate Centre at Vancouver General Hospital, 2660 Oak Street, V6H 3Z6, Vancouver, BC, Canada; 3 Department of Biopathology and Diagnostic Imaging, Tor Vergata University of Rome, Viale Oxford, 81-00133, Rome, Italy

## Abstract

**Background:**

The androgen receptor is a ligand-induced transcriptional factor, which plays an important role in normal development of the prostate as well as in the progression of prostate cancer to a hormone refractory state. We previously reported the identification of a novel AR coactivator protein, L-dopa decarboxylase (DDC), which can act at the cytoplasmic level to enhance AR activity. We have also shown that DDC is a neuroendocrine (NE) marker of prostate cancer and that its expression is increased after hormone-ablation therapy and progression to androgen independence. In the present study, we generated tetracycline-inducible LNCaP-DDC prostate cancer stable cells to identify DDC downstream target genes by oligonucleotide microarray analysis.

**Results:**

Comparison of induced DDC overexpressing cells versus non-induced control cell lines revealed a number of changes in the expression of androgen-regulated transcripts encoding proteins with a variety of molecular functions, including signal transduction, binding and catalytic activities. There were a total of 35 differentially expressed genes, 25 up-regulated and 10 down-regulated, in the DDC overexpressing cell line. In particular, we found a well-known androgen induced gene, *TMEPAI*, which wasup-regulated in DDC overexpressing cells, supporting its known co-activation function. In addition, DDC also further augmented the transcriptional repression function of AR for a subset of androgen-repressed genes. Changes in cellular gene transcription detected by microarray analysis were confirmed for selected genes by quantitative real-time RT-PCR.

**Conclusion:**

Taken together, our results provide evidence for linking DDC action with AR signaling, which may be important for orchestrating molecular changes responsible for prostate cancer progression.

## Background

Prostate cancer is the most commonly diagnosed invasive male cancer in North America and in other Western countries [1]. In most cases prostate cancer begins as an androgen-dependent tumor that undergoes clinical regression in response to pharmacological and surgical strategies that reduce testosterone concentration. This form of therapy is generally used to treat advanced cancer or those that recur after radiation or surgical procedures to remove the primary cancer. Despite androgen withdrawal therapy most patients develop lethal androgen-independent (AI) tumors [2,3]. At present, no effective therapy is available for this latter group of patients [4]. The underlying molecular mechanism involved in androgen-independent prostate cancer and the therapies aimed at this are the active areas of current research.

The actions of androgen within the prostate are mediated by the androgen receptor (AR), a member of the nuclear receptor family of ligand-activated transcription factors [5]. Upon binding hormone, AR binds to androgen response elements in androgen receptor-responsive promoters, recruits multiple coregulators, and activates transcription of androgen-regulated genes involved in cell growth and survival [4,6,7]. In the majority of AI tumors, AR continues to be expressed and seems to be activated under androgen-depleted conditions [8]. Alterations in AR or the AR signaling pathway are potential explanations for progression to androgen independence [9,10]. A large number of coactivators and corepressors involved in the regulation of AR-driven transcription have been identified [11]. They function as signaling intermediaries between AR and general transcriptional machinery. Furthermore, an increase in coactivator levels has been shown in AI disease [12-16]. Coactivator proteins have been shown to enhance the activity of AR through a variety of mechanisms, including use of alternative ligands, sensitization of the receptor to lower levels of androgens, and ligand-independent activation [14,17].

Using the repressed transactivator (RTA) yeast two-hybrid system, we previously identified a novel AR-coactivator protein, L-dopa decarboxylase (DDC) [18], also referred to as aromatic L-amino acid decarboxylase (AADC). DDC is responsible for decarboxylating both L-dopa and L-5-hydroxytryptophan into dopamine and serotonin, respectively [19]. The human gene encoding the L-dopa decarboxylase enzyme, referred to as *DDC*, maps to chromosome band 7p11 and is composed of 15 exons spread out over at least 85 kb of genomic DNA [20]. DDC is widely distributed in neural tissues, where it plays a neuron-specific role as a neurotransmitter biosynthetic enzyme, and in non-neuronal tissues (adrenals, kidney, liver, gastrointestinal tract and lungs), where it acts as a non-specific decarboxylating enzyme and may have other undetermined functions [21]. Our recent studies using tissue microarrays and dual immunofluoresence indicate that in prostate cancer, DDC is not only a neuroendocrine (NE) marker, but is also co-expressed with AR in a subset of NE tumor cells [22]. DDC-positive prostate cancer cells show a dramatic increase in number after extended periods of neoadjuvant hormone withdrawal (> 6 months) and in metastatic tumors that have progressed to the AI phenotype [22]. The enhancement of AR transactivation by DDC is likely restricted to the AR-positive subset of NE cells. The mechanism of DDC-mediated regulation of AR signaling in prostate carcinogenesis remains unknown, but may involve sensitization of AR to limiting concentration of androgen [18].

In the past few years, newly developed technologies such as gene microarrays [23] have enabled the determination of molecular differences between normal and transformed cells at the genome wide-level. Microarrays have been used to study androgen regulated genes involved in the development of prostate cancer [24], and to characterize molecular function of AR or other steroid receptor interacting proteins [25-27]. In particular, microarray analysis has been used to better define the molecular function of Ebp1 protein, an AR corepressor [25], and to study AIB1 protein, a steroid receptor coactivator [27], as well as to characterize a novel modulator of AR activity, the male germ cell-associated kinase (MAK) [26]. These studies suggest that microarray analysis is a useful means for studying the effects on gene expression of steroid receptor interacting proteins. Here, in an effort to better understand the molecular function of DDC as a coactivator of AR-mediated signaling and to identify novel targets of prostate carcinogenesis, we evaluated the effects of regulated DDC expression in an inducible manner in LNCaP cells using gene microarray analysis.

## Results

### Inducible expression of DDC in LNCaP human prostate cancer cells

**Figure 1:**
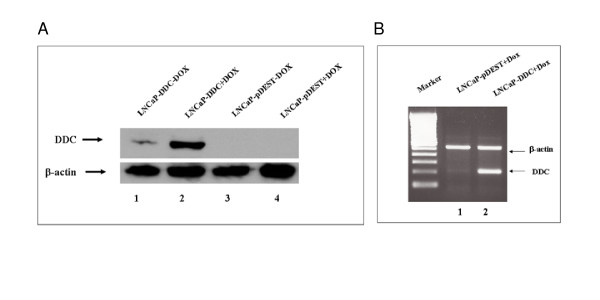
**LNCaP-DDC cell line displays regulated DDC expression with Dox treatment**. Shown are the results from (**A**) Western blot and (**B**) semiquantitative RT-PCR analysis of the expression of DDC protein and mRNA, respectively. In **A, **LNCaP-DDC and LNCaP-pDEST (vector control) cells were treated for 48 hours under mock-induced (-Dox) and Dox-induced (+Dox) conditions before protein lysate preparation. No visible expression was detected in the LNCaP-pDEST control cells regardless of Dox treatment status (lines 3–4). A 6 fold increased DDC expression level was detected in the Dox-induced LNCaP-DDC cells compared to the mock-induced cells (lines 1–2), after β-actin normalization. In **B, **total cellular RNA was isolated from LNCaP-DDC and LNCaP-pDEST cells after 48 hours of Dox treatment. After reverse transcription, PCR was performed with DDC and β-actin specific primers. The up-regulation of the 209-bp DDC-specific band was detected in LNCaP-DDC cells (line 2). A 100 bp DNA ladder (Promega) was used for size markers.

The identification of AR-regulated genes that are affected by DDC overexpression may provide important clues regarding the biology of this catecholamine synthesis enzyme and its influence on AR function. Toward this end, the change in gene expression of androgen-regulated genes caused by sustained DDC overexpression were analyzed *in vitro *by the generation of LNCaP cells stably expressing Dox-inducible DDC. We used human androgen-dependent prostate cancer cells (LNCaP), since our primary goal was to assess the co-activation function of DDC, known to enhance AR activity through an androgen-dependent mechanism [18]. To verify the effects of Dox treatment on expression of the *DDC *gene, LNCaP cells, stably expressing Dox-inducible DDC (LNCaP-DDC) or the vector control (LNCaP-pDEST) were treated for 48 hours under mock-induced (-Dox) and Dox-induced (+Dox) conditions. With Dox treatment, we observed an optimal 6-fold increase in DDC protein levels when compared with mock-induced (-Dox) LNCaP-DDC cells. No detectable DDC protein was found under the Dox-induced (+Dox) or mock-induced (-Dox) condition in the LNCaP-pDEST cell line, confirming the fact that endogenous DDC protein expression levels in this cell line are below detection when directly compared to ectopic overexpression (Figure 1A) [18]. To investigate the extent of DDC overexpression at the RNA level, we compared by RT-PCR the mRNA levels in DDC overexpressing cells, with the vector control cells after Dox stimulation. In the LNCaP-DDC cells, higher levels of DDC mRNA were observed than in the vector control LNCaP-pDEST cells, where only a faint band was detected (Figure 1B).

### Influence of DDC on the androgen-regulated gene expression profile

**Figure 2:**
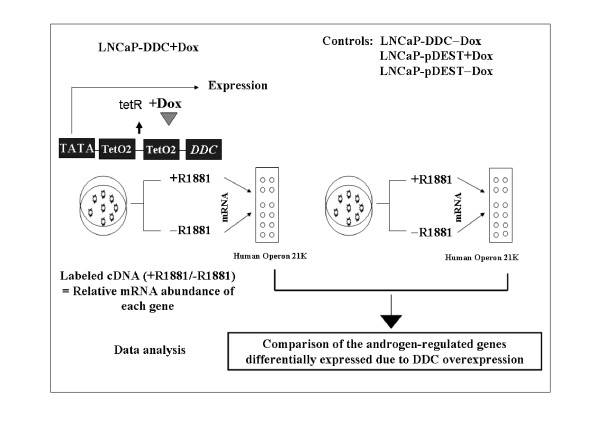
**Microarray analysis of DDC-regulated genes**. Experimental outline of microarray studies to identify DDC downstream targets using LNCaP-DDC stable and control LNCaP-pDEST cells. In the presence of Dox (*grey triangle*, + Dox) the tetracycline Tet repressor (tetR) is released from the TetO2 sequence in the promoter of the lentiviral construct containing the *DDC *gene. The dissociation of the tetR allows induction of transcription for the gene of interest. LNCaP-DDC and LNCaP-pDEST (vector control) cell lines were plated in medium containing 5% charcoal-stripped serum and incubated overnight. The next day, cells were treated for 48 hours under mock-induced (-Dox) and Dox-induced (+Dox) conditions, 24 h of which was in the presence or absence of 0.1 nM R1881. Total RNA samples were isolated from each condition, labeled and hybridized on the microarrays. The relative mRNA abundance of each gene was calculated as a ratio between hormone-treated (+R1881) and hormone-untreated (- R1881) samples. The comparison of the expression data obtained from LNCaP-DDC+Dox stable cells (*left*) with the expression data from the three LNCaP control cells (*right*: LNCaP-DDC-Dox, LNCaP-pDEST- Dox, and LNCaP-pDEST+Dox) yielded the identification of genes that are androgen- and DDC-regulated.

**Figure 3:**
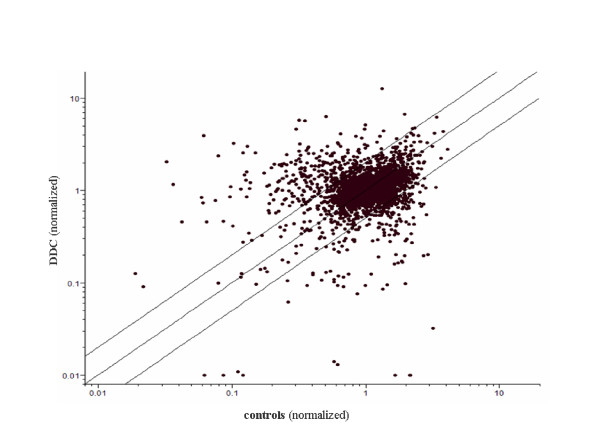
**Scatter plot of entire gene set considered expressed (p values = 0.05) in the microarray analysis**. The position of each dot on the scatter plot corresponds to the normalized average signal intensity (log scale) of a single gene. The normalized average signal intensity under the DDC overexpression conditions are shown on the x and y axes (controls = no DDC overexpression and DDC = DDC overexpression). The middle line indicates values that represent a DDC/controls ratio of 1.0 (similar levels of expression in both cell lines). The outer lines represent a DDC/controls ratio of 2.0 (upper line; 2-fold greater expression in DDC compared to controls) and of 0.5 (lower line; 2-fold greater expression in controls compared to DDC).

The availability of the LNCaP-DDC cell line afforded us an opportunity to study the influence of DDC overexpression on global gene expression using the Human Operon 21 K oligonucleotide array. Our goal in this study was to identify which genes among the AR-regulated genes, are affected by DDC overexpression. We prepared total RNA from DDC overexpressing cells (LNCaP-DDC) and from the LNCaP control cell lines (LNCaP-DDC-Dox, LNCaP-pDEST- Dox, and LNCaP-pDEST+Dox) after 48 h treatment with or without Dox, 24 h of which was in the presence or absence of R1881 synthetic hormone (Figure 2). Independent probe synthesis from different batches of RNA and hybridizations were performed in duplicate. To account for dye bias a dye swap was also performed. To identify the androgen-regulated genes in both LNCaP-DDC and LNCaP control cells, total RNA isolated from hormone treated (+R1881) samples was combined with total RNA isolated from hormone-untreated (-R1881) samples and the relative abundance of each gene was calculated as a ratio between hormone-treated and hormone-untreated samples. The data set was normalized, and a filter was applied to select only those genes whose expression level was significant (*p *≤ 0.05) in at least 1 out of the 2 conditions. A total of 3,127 genes were identified, and presented as a scatter correlation plot in Figure 3.

Genes that showed significant expression levels across cell types (*p *≤ 0.05) were further analyzed to characterize those genes whose expression levels were increased or repressed by at least 2-fold during hormone treatment (± R1881). Genes showing an expression level > 0.5 and < 2 were classified as unchanged and not considered further. In this study, we focused on those genes that were hormone up-or down-regulated in both LNCaP-DDC and LNCaP control cells.

**Figure 4:**
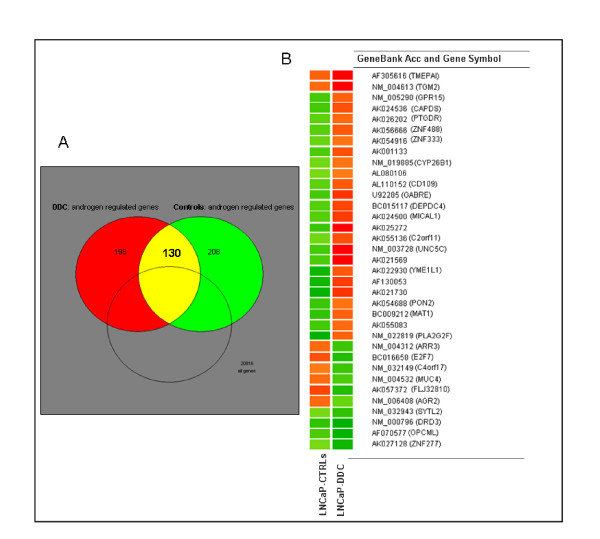
**Androgen-regulated and DDC-regulated genes. A) **A Venn diagram analysis showing in yellow, the genes (130) with two fold induction in response to R1881 treatment in DDC overexpressing and control cells. The genes up- and down-regulated only in DDC overexpressing cells are represented in red (DDC; *left*) and the genes up- and down-regulated only in the control cells are represented in the green (controls; *right*). **B) **Reported here are 35 androgen-regulated genes differentially expressed at least 2-fold in DDC overexpressing cells (LNCaP-DDC) compared with the controls cells (LNCaP-CTRLs). The colors represent the ratio of gene expression levels in each cell line after R1881 treatment (red = hormone up-regulated and green = hormone down-regulated).

Among the androgen-regulated genes we identified, using Venn diagram analysis, 130 genes that were androgen-regulated in LNCaP-DDC and LNCaP control cells (Figure 4A). The hormone induction response of these 130 genes was substantiated by the altered expression of the classically androgen-regulated genes, such as *PSA*, *FKBP5, NKX3A, TMEPAI, KLK2, ODC1*, and *TMPRSS2 *(data not shown). Comparison of LNCaP-DDC and LNCaP control cells revealed a number of changes in the expression profile of these androgen-regulated genes. Out of the 130 genes, 35 were differentially regulated as shown in Table 1. Of these, 25 genes were up-regulated at least two fold and 10 were down-regulated by at least two fold with DDC overexpression. In particular, among the set of 35 genes, we could identify four different responses to hormone treatment and DDC overexpression: *i*) 2 genes were hormone and DDC up-regulated *ii*) 4 genes were hormone and DDC down-regulated *iii*) 23 genes were hormone down-regulated in control cells and hormone up-regulated in DDC overexpressing cells *iv*) 6 genes were hormone up-regulated in control cells and hormone down-regulated in DDC overexpressing cells (Figure 4B).

**Table 1:** Differentially expressed genes in response to DDC overexpression*. The genes shown here are those whose expressions are increased (≥ 2) or decreased (≤ -2).

**Genbank Acc**	**Gene Symbol & Name**	**DDC/CTRLs**
*Binding*		
BC016658	*E2F7; E2F transcription factor 7*	-13.60
AK024500	*MICAL1; microtubule associated monoxygenase*	+7.92
BC009212	*MTA1; metastasis associated 1*	+9.50
NM_032943	***SYTL2; synaptotagmin-like 2***	**-2.02**
AK027128	*ZNF277; zinc finger protein 277*	-3.88
AK054916	*ZNF333; zinc finger protein 333*	+4.67
AK056666	*ZNF488; zinc finger protein 488*	+6.76
*Binding and catalytic activity*		
NM_019885	*CYP26B1; cytochrome P450, family 26, subfamily B, polypeptide 1*	+4.29
NM_022819	*PLA2G2F; phospholipase A2, group IIF*	+30.10
NM_004613	***TGM2; transglutaminase 2 ***	**+2.10**
AK022930	***YME1L1; YME1-like 1 (S. cerevisiae)***	**+21.40**
*Binding and signal transducer activity*		
NM_000796	***DRD3; dopamine receptor D3***	**-4.28**
U92285	*GABRE;Gamma-aminobutyric acid (GABA) A receptor, epsilon*	+10.02
NM_004532	*MUC4; mucin 4, tracheobronchial*	-6.39
AF070577	*OPCML; opioid binding protein/cell adhesion molecule-like*	-3.87
NM_003728	***UNC5C; unc-5 homolog C (C. elegans)***	**+18.48**
*Catalytic activity*		
AK055136	*C2orf11; chromosome 2 open reading frame 11*	+5.79
AK054688	*PON2; paraoxonase 2*	+6.99
*Signal transducer activity*		
NM_005290	*GPR15; G protein-coupled receptor 15*	+7.47
AK026202	***PTGDR; prostaglandin D2 receptor (DP)***	**+5.26**
*Unclassified*		
AL110152	*CD109; CD109 antigen (Gov platelet alloantigens)*	+6.20
NM_006408	*AGR2; anterior gradient 2 homolog (Xenopus laevis)*	-5.31
NM_004312	*ARR3; arrestin 3, retinal (X-arrestin)*	-8.08
NM_032149	*C4orf17; chromosome 4 open reading frame 17*	-6.81
AK024536	*CADPS; Ca2+-dependent secretion activator*	+8.45
BC015117	*DEPDC4; DEP domain containing 4*	+7.83
AK057372	*FLJ32810; hypothetical protein FLJ32810*	-14.32
AF305616	***TMEPAI; transmembrane, prostate androgen induced RNA***	**+2.06**
AK025272	*Unkown*	+15.27
AK021730	*Unkown*	+31.81
AF130053	*Unkown*	+23.33
AL080106	*Unkown*	+4.04
AK021569	*Unkown*	+16.22
AK001133	*Unkown*	+7.59
AK055083	*Unkown*	+6.91

*Bold indicated genes investigated further by RT-PCR

Gene ontology classification of these 35 differentially expressed genes was performed by using GeneBank accession number, Operon ID, and annotation tools database available on-line [28,29]. The gene sets were classified according to their putative main molecular function. While the list of 35 genes contained 15 unclassified transcripts, of the remaining 20, ontological molecular function analyses revealed signal transducer, binding, and catalytic activity as the predominant divergences between DDC overexpressing cells and controls (Table 1). Gene ontology classifications with overlapping gene lists were combined. The signal transducer classification defines those transcripts that mediate the transfer of a signal from the outside to the inside of a cell by a means other than the introduction of a signal molecule itself into the cell. The binding activity category defines those transcripts encoding for proteins capable of selective, often stoichiometric, interaction with one or more specific sites on another protein. The catalytic activity group refers to those proteins able to perform the catalysis of a biochemical reaction. Further annotation analysis of the transcripts involved in signal transduction, subclassified four genes into the G-protein-coupled receptors (GPCRs) signaling pathway (*DRD3, PTGDR, GPR15*, and *GABRE*). Interestingly, we found a 4.3-fold down-regulation of the *DRD3 *gene in DDC overexpressing cells when compared with controls. The *DRD3 *gene encodes the D3 subtype dopamine receptor which binds dopamine, one of the enzymatic products of DDC [30]. The D3 receptor, along with the other GPCRs, are known to modulate a plethora of signal transduction pathways that can cross-talk with known AR activation pathways [4]. Among the genes up-regulated by DDC overexpression, there was a well established androgen regulated *TMEPAI *gene [31,32] (Table 1). Another gene up-regulated by DDC overexpression was *TGM2*, which belongs to the transglutaminase (hTGP) enzyme family (EC 2.3.2.13) [33]. Interestingly, the expression of one of these family members, prostate transglutaminase enzyme (TGM4), has been shown to be androgen-regulated [34]. *SYTL2 *was among the androgen down-regulated genes, and has been previously shown to be differentially regulated by AR reduction in prostate cancer cells [35]. Overall, our results indicated that 35 genes were differentially expressed after induced overexpression of DDC in LNCaP cells, including genes known to be up- or down-regulated by AR.

### Verification of microarray results by quantitative RT-PCR

To verify the results of our microarray analyses, we selected seven representative genes from the list in Table 1 and determined their expression profiles by quantitative real-time RT-PCR. *TMEPAI, SYTL2, TGM2, DRD3*, and *PTGDR *were chosen because of the above consideration, whereas *UNC5C *and *YME1L1 *genes were selected based on the large magnitude of change (Table 1) and *GAPDH *was used as an endogenous control. The same RNA samples used for the microarray hybridization were examined. For simplicity we did not consider the mock-induced (-Dox) samples, but compared the RNA extracted from LNCaP-DDC Dox-induced cells (+Dox) versus the RNA extracted from the empty vector LNCaP-pDEST Dox-induced cells (+Dox). Although the magnitudes of up-or down-regulation of the *TMEPAI, SYTL2, TGM2, DRD3, YME1L1 *and *PTGDR *genes in the array analysis were different than that observed by real time RT-PCR, there was a similar trend in the changes with respect to both R1881 treatment and DDC overexpression (Table 2). A constant level of *GAPDH *was observed. Unfortunately no voluble template was obtained for the *UNC5C *gene. Overall, the above results verify the data observed in our microarray studies.

**Table 2:** Confirmation of microarray findings by real-time RT-PCR*

	**R1881 ± **		**R1881 ± **			
			
**Symbol**	**ARRAY DDC**	**ARRAY controls**	**RT-PCR DDC**	**RT-PCR control**	**ARRAY DDC/controls**	**RT-PCR DDC/control**
***TGM2***	**4.7**	**2.2**	**36.7**	**25.2**	+2.1	+1.5
***TMEPAI***	**4.8**	**2.3**	**17.8**	**10.6**	+2.1	+1.7
***PTGDR***	**2.2**	*0.4*	**1.5**	*0.5*	+5.3	+3.0
***YME1L1***	**2.6**	*0.1*	**1.3**	*0.8*	+21.4	+2.5
***SYTL2***	*0.3*	*0.5*	*0.2*	*0.3*	- 2.0	- 1.5
***DRD3***	*0.1*	*0.3*	*0.5*	*0.9*	- 4.3	- 1.8

* In **bold **are represented the fold changes of hormone up-regulated genes and in *italic *the fold changes of hormone down-regulated genes, after R1881 treatment. The symbols +/- indicate the fold induction (+) or repression (-) due to DDC overexpression.

## Discussion

Our laboratory has shown that DDC is a coactivator of AR [18] and a neuroendocrine marker of prostate cancer that increases in expression during hormone-ablation therapy and after progression to AI [22]. Coregulator proteins play a crucial role in modulating transactivation of AR and consequently may be important in regulating aberrant activity of AR during prostate cancer progression. In this study, we explored the effect of overexpression of DDC on the gene expression profile of an androgen-dependent prostate cancer cell line. We employed a tetracycline-regulated system to inducibly overexpress DDC and screen for potential downstream target genes in LNCaP cells using an oligonucleotide array with 21 K individual human genes. In particular, we first selected a list of genes that were androgen-regulated regardless of DDC status. To this end, in both DDC overexpressing and control cells, we selected all the genes that were significantly expressed (*p *≤ 0.05) and which displayed at least a 2 fold increased or decreased expression due to hormone treatment (± R1881). Our interest was then focused on the identification of those genes that were differentially regulated by DDC overexpression.

Comparison of DDC overexpressing cells (LNCaP-DDC) versus controls cells revealed a number of changes in the expression of androgen-regulated transcripts encoding proteins with a variety of molecular functions, including signal transduction, binding and catalytic activities. Although the classically androgen-regulated gene, prostate-specific antigen (PSA), was on the chip and was found in the hormone up-regulated genes group, it did not show any significant difference in gene expression levels when DDC was overexpressed. This may be due to the possibility that PSA expression is not DDC-specific or long-term (> 2 days) overexpression of DDC, may be required to increase PSA expression. This was not entirely unexpected since most classical coactivators, such as SRC/p160 family members, have not been reported to increase androgen-induced expression of endogenous PSA in LNCaP cells.

In this study, 25 up-regulated and 10 down-regulated genes were identified in DDC overexpressing cells compared with control cells (Table 1). Real-time RT-PCR confirmed and validated the microarray gene expression data. The difference in magnitude observed between microarray findings and quantitative RT-PCR is likely due to different technique efficiency and/or primers specificity (Table 2). Among the genes that were hormone and DDC up-regulated, there was a well established androgen regulated gene,*TMEPAI *[31]. In a previous study, evaluation of *TMEPAI *(also referred to as *PMEPA1*) expression in LNCaP cells demonstrated induction by androgen in a time- and dose-dependent manner. Interestingly, the authors also showed that *TMEPAI *was overexpressed in AI tumors when compared with androgen sensitive tumors [31]. Another gene found to be hormone and DDC up-regulated is the *TGM2 *gene, which belongs to the transglutaminases (TGases) family (EC 2.3.2.13) [33]. The transglutaminases are calcium-dependent enzymes catalyzing the post-translational cross-linking of proteins. Even though no direct androgen regulation has been reported for the *TGM2 *gene, another transglutaminase enzyme (*TGM4*) has been found to be androgen regulated in human prostate cancer cell lines [34]. Androgen and DDC induced expression of *TMEPAI *and *TGM2 *may represent direct evidence of the androgen-dependent co-activation function of DDC on AR.

Among the hormone and DDC down-regulated genes was the *SYTL2 *gene, which was previously shown to be positively regulated by AR reduction in androgen-ablated prostate cancer cells [35]. In particular, a 2 fold up-regulation of the *SYTL2 *gene was found after 48 h reduction of AR [35], demonstrating that *SYTL2 *is normally an androgen-repressed gene. In our study, overexpression of DDC further reduced the expression of *SYTL2*, suggesting that this AR-binding protein may also augment the repressive transcriptional function of AR. The *SYTL2 *gene encodes for the vesicular transport protein synaptotagmin 2, which regulates exocytosis of synaptic vesicles and appears to serve as a calcium sensor to trigger neurotransmitter release [36]. A possible explanation of this finding could be related to the increased synthesis of neurotransmitters produced by DDC inside the cell, and subsequent negative feedback on these vesicular transporter related proteins.

Among the genes differentially regulated in DDC overexpressing cells, were four genes encoding GPCR proteins (*DRD3, PTGDR, GPR15*, and *GABRE*). The GPCRs share significant structural homology [37,38] and are known to modulate numerous of signal transduction pathways that can cross-talk with known AR activation pathways [4]. A general model for GPCR activity has been postulated where GPCRs are in equilibrium between active and inactive states, and that interaction with a GPCR agonist, stabilizes a conformational change in these receptors which in turn promote signal generation inside the cell [39]. Recently, it has been shown that transition of prostate cancer to the AI stage is associated with increased expression of GPCRs [40-42]. Furthermore, *in vitro *stimulation of endogenous GPCRs (e.g. LPA, B1R) induces mitogenic signaling and growth of AI prostate cancer [42-44]. Prostate cancers also express elevated levels of GPCR ligands, which may contribute to progression of disease [40,41,45]. Therapies targeting GPCRs represent the single largest drug class [46], suggesting that they may be effective in limiting pathologic growth of the prostate. Further studies are required to establish any relationships between prostate cancer progression and increased expression levels of *PTGDR, GPR15*, and *GABRE *genes. In contrast, the *DRD3 *gene was found to be down-regulated in DDC overexpressing cells when compared with control cells. The *DRD3 *gene encodes the D3 subtype dopamine receptor for which the DDC neurotransmitter product, dopamine, is an agonist. D3 receptor is classified as a member of the D2-like dopamine receptor family, which also includes the D2 and D4 dopamine receptor subtypes [47,48]. Interestingly, it has been shown that D2-like receptors can modulate many signal transduction pathways (e.g. MAPK, PKA, PKC) [49-51] that are also known to stimulate AR activation [4,52,53]. Altered regulation of this receptor in prostate cancer cells could lead to indirect activation of the AR signaling pathway. Since D2-like receptors can mediate inhibition of cAMP and PKA signaling [51], one can speculate that in the presence of dopamine, decreased expression of the D3 receptor subtype could reduce its inhibitory effect on the cAMP and PKA pathways when DDC is overexpressed. This may result in high level activation of PKA signaling that can enhance AR activity [4]. Overall, the altered expression of GPCRs by DDC may result in increased mitogenesis and growth of prostate tumors.

## Conclusion

This study demonstrates that overexpression of the *DDC *gene in the LNCaP cells leads to differential expression of a total of 35 genes. More detailed studies examining the association between the AR-DDC interaction and these genes are necessary to better understand the functional relationships. Potentially all these putative hormone-regulated genes are directly or indirectly downstream targets of DDC and they may be important for orchestration of molecular changes that are responsible for prostate cancer progression. Also since DDC expression increases with long-term neo-adjuvant hormone therapy (> 6 months) and in metastatic tumors that have progressed to the AI phenotype [22], future studies could utilize castrated (hormone-deprived) mice in the LNCaP xenograft model as an *in vivo *experimental system for monitoring the potential effects of DDC on the expression profile of genes associated with growth, regression, and progression to AI.

## Methods

### LNCaP cells expressing tetracycline-inducible DDC

LNCaP cells stably expressing tetracycline-inducible DDC (LNCaP-DDC) were generated using ViraPower T-REx Lentiviral Expression System and Gateway Technology vectors, according to the manufacturer's protocol (Invitrogen) (Wafa LA, 2007 *manuscript in preparation*). Briefly, 3 μg of each lentiviral vector, pLenti4/TO/V5-DEST carrying the *DDC *gene or the empty vector pLenti4/TO/V5-DEST and the pLenti6/TR containing the *TetR *gene, together with 9 μg of the ViraPower packaging mix, were transfected into 293T cells, using Lipofectamine 2000 reagent (Invitrogen, Carlsbad, CA). The DNA- Lipofectamine 2000 complexes diluted in Opti-MEM I Medium (Gibco-BRL) were allowed to form for 20 min at room temperature before addition to 293T cells. Cells were maintained for 24 hours at 37°C and 5% CO_2 _before removing the media containing the DNA-Lipofectamine 2000 complexes and replacing with DMEM media (10% FBS, 2 mM L-glutamine, 0.1 mM MEM Non-Essential Amino Acids, 1% penicillin/streptomycin, and 1 mM MEM Sodium Pyruvate).

Resulting retroviral particles were harvested by removing medium 72 hours after transfection and used to generate a stably co-transduced LNCaP cell lines. Two cell lines were created: the LNCaP-DDC line, which expresses tetracycline-inducible DDC, and the LNCaP-pDEST line, which contains the empty vector control and the *tetR *gene. To induce tetracycline-regulated DDC expression 1 μg/ml of doxycycline hyclate (Dox) (Sigma-Aldrich) was added to the cell culture media.

### RNA isolation and expression profiling

LNCaP-DDC, and LNCaP-pDEST cell lines were plated in 10-cm plates (2 × 10^6 ^per plate) in RPMI 1640 containing 5% charcoal-stripped serum (CSS; HyClone, VWR, West Chester, PA). When the cells reached 60% confluence, they were seeded in presence of 1 μg/ml of Dox (Dox-induced) or in the absence of Dox (mock-induced). After 24 hours the cells were stimulated with or without 0.1 nM of synthetic androgen (± R1881) for an additional 24 hours. Total RNA was extracted using Trizol reagent by following the manufacturer's instructions (Invitrogen). Total RNA from each hormone-treated sample (+R1881) was compared with that of hormone-untreated sample (-R1881) on the same microarray slide. Two independent biological replicates were assayed for each sample and a dye swap was performed to account for dye bias. Microarrays of 21,521 (70-mer) human oligos representing 21,521 genes (Operon Technologies, Inc., Alameda, CA) printed on aminosilane coated microarray slides (Matrix Technologies; Hudson, NH) were supplied by the Array Facility of The Prostate Centre at Vancouver General Hospital. Microarrays were hybridized with 10 μg of total RNA from duplicate samples of LNCaP-DDC or LNCaP-pDEST cells treated with or without (±) Dox, and with or without (±) R1881, using the 3DNA Array 350™ Expression Array Detection Kit and according to the manufacturer's instructions (Genisphere, Hatfield, PA) (Figure 2). The arrays were immediately scanned on a Scan Array Express Microarray Scanner (Perkin Elmer). Signal quality and quantity were assessed using ImaGene 7.0 software (BioDiscovery, San Diego, CA).

### Western blot analysis

The DDC antibody was purchased from Chemicon (Chemicon Inc., Temecula, CA) and the polyclonal antibody to actin was obtained from Sigma (Sigma Chemical Co., St. Louis, MO). LNCaP-DDC and LNCaP-pDEST cells were plated in six-well plates (3 × 10^5 ^per well), treated with Dox as described above and immunoblotted as previously reported [18]. Total cell protein (50 μg), measured by BCA™ Protein Assay (PIRCE, Rockford, IL), was used for immunoblotting. Band intensity was quantified using a Bio-Rad Gel Doc 2000 software (Bio-Rad Laboratories, USA).

### Bioinformatics analysis

Raw signal data files obtained with ImaGene 7.0 software were subsequently analyzed on GeneSpring 7.2 software (Silicon Genetics, Redwood City, CA) for profiling significant changes in gene expression. The fluorescent signal ratios (+R1881/-R1881) were subjected to Lowess normalization with background correction. Experimental error was based on replicated dye pair values. Comparison analysis of the gene expression data from LNCaP-DDC+Dox cells, and LNCaP-DDC-Dox, LNCaP-pDEST-Dox, and LNCaP-pDEST+Dox cells, treated with or without R1881, was conducted to first identify androgen-regulated genes in all experimental conditions, and then the differentially regulated genes due to DDC overexpression.

Only genes with a *p *value of ≤ 0.05 in at least one out of two conditions were analyzed further. Data was transformed to log ratio (Log_10_) for display and analysis. All genes showing a normalized expression value ≥ 2 or ≤ 0.5 were classified as androgen up- or down-regulated, respectively. Genes showing a normalized expression value between 0.5- and 2- were classified as unchanged and not considered further. Lists of androgen-regulated genes (both up-regulated and down-regulated) were created for each of the cell lines and were compared by Venn diagram analysis. Genes were considered differentially expressed as a result of DDC overexpression if normalized values from induced LNCaP-DDC cells were at least 2-fold greater or 2-fold less than those from the control cells (LNCaP-DDC-Dox, LNCaP-pDEST- Dox, and LNCaP-pDEST+Dox). Functional classifications were based on gene ontology (GO) annotation obtained through the *GeneTools *database [28,29].

### Real-time quantitative RT-PCR and semiquantitative RT-PCR

Complementary DNA (cDNA) for real time PCR and semiquantitative RT-PCR was made using 2 μg of total RNA treated with RNase-free DNase according to the manufacturer's instructions (Promega). First-strand cDNA was synthesized using random hexamers (Perkin-Elmer Applied Biosystems, Branchburg, NJ) with 20U of Moloneymurine leukemia virus reverse transcriptase, M-MLV (Invitrogen). The ABI PRISM 7700 Sequence Detection System (Perkin-Elmer Applied Biosystems, Foster City, CA) was used for real time monitoring of PCR amplification of cDNA. The forward and reverse primers used for the real time RT-PCR are listed in Table 3. All primers were selected by PRIMER EXPRESS v.3 software and were commercially synthesized by Integrated DNA Technologies laboratories (IDT, Coralville IA). A SYBR green PCR kit was used following the manufacturer's instructions and the analyses were performed in triplicate (Invitrogen). In brief, RT-PCR amplification mixtures (25 μl) containing 25 ng template cDNA, 2x SYBR Green I Master Mix buffer (12.5 μl), and 300 nM forward and reverse primer was prepared. Target mRNA values were normalized using *GAPDH *mRNA as an internal control. A comparative threshold cycle (Ct) was used to determine gene expression relative to a calibrator. For each sample, the Ct values were calculated using the formula ΔC_T _= C_t sample_- C_t GAPDH_. To determine relative expression levels, the following formula was used ΔΔC_T _= ΔC_T sample_- ΔC_T calibrator _and the value used to indicate relative gene expression was calculated using the formula 2^-ΔΔCT^. Primers for the semiquantitative PCR were synthesized by Integrated DNA Technologies laboratories (IDT, Coralville IA); *DDC*, sense 5'- ACA CCA TGA ACG CAA GTG AA-3' and antisense 5'- CAC CCC AGG CAT GAT TAT CT-3'. The PCR products (209 bp) were resolved by electrophoresis using 1.5% agarose gels containing ethidium bromide.

**Table 3:** Primers for real time RT-PCR analysis

**Gene**	**Forward primer**	**Reverse primer**
***TMEPAI***	TGCCGTTCCATCCTGGTT	AGACAGTGACAAGGCTAGAGAAAGC
***YME1L1***	GAGCTTGGACACAACCGATAACT	CCGCAGTGTACAGGGATTGA
***SYTL2***	TCTGCCTTGAGAAAACAAACAGTT	GCCAGTGGGTGGCACTAAAA
***UNC5C***	GGCCGTCCAGGTGAATCA	TGCATTCTTGCCTGTGAAGTG
***TGM2***	TCTCTGGGCCTTTGTTTCCTT	GATCCTTGGAGATGAGCTGGTT
***PTGDR***	TCAGGACTCCAAGGTGCAAAG	TCTGGCTGGAGGTCTTGAGATC
***DRD3***	GAATTCCCTGAGTCCCACCAT	CCATTGCTGAGTTTTCGAACTTC
***GAPDH***	GAAGGTGAAGGTCGGAGT	GAAGATGGTGATGGGATTTC

## Competing interests

The author(s) declare that they have no competing interests.

## Authors' contributions

K.M. performed the study and drafted the manuscript; L.A.W. helped to design the study and to draft the manuscript; H.C. helped to perform the study; G.N. critically commented on the drafted manuscript; C.C.N participated in the design of the study and critically commented on the drafted manuscript; P.S.R. provided overall direction for the project and revised the final version of the manuscript; G.N., C.C.N., and P.S.R obtained funding for the project. All authors read and approved the final manuscript.
